# Supplementary material for: Investigating differences in the implementation and experience of the Enhanced Health in Care Homes Framework in England: a qualitative protocol for the Understanding Variation in Admissions from Care Homes (UVAC) study
Source: BMJ Open. 2026 May 28;16(5):e112414. doi: 10.1136/bmjopen-2025-112414 (PMC13223941; doi:10.1136/bmjopen-2025-112414)

Hospital Admissions – ICB – First Cohort

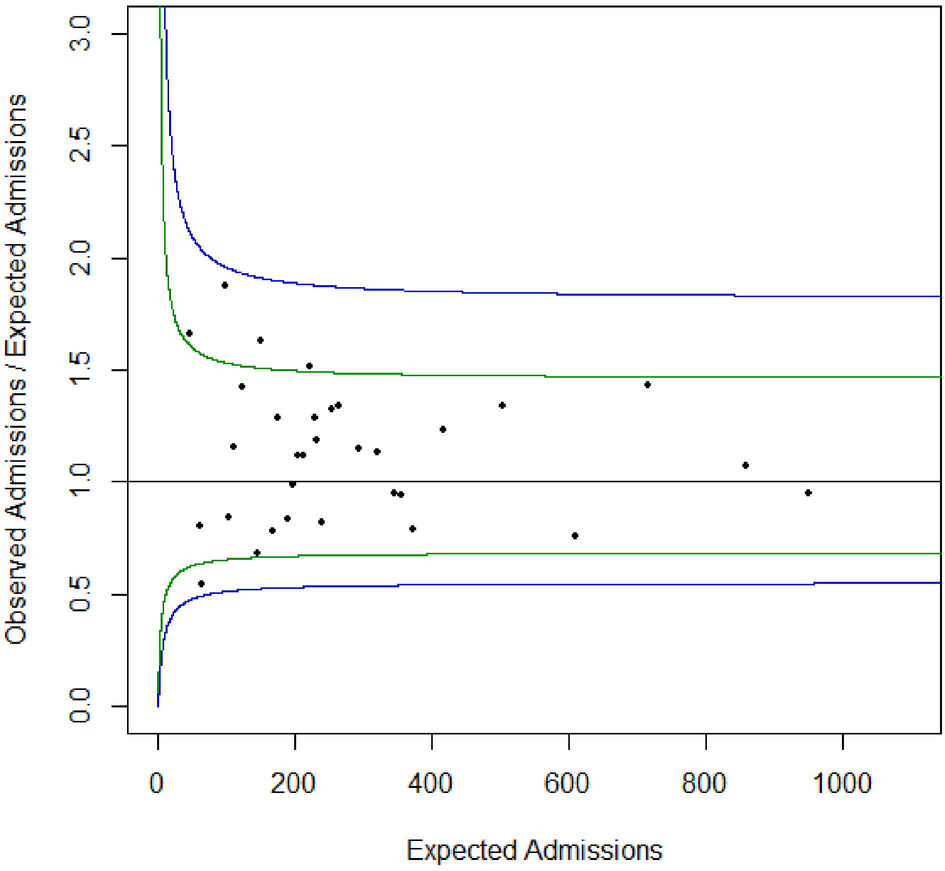

Hospital Admissions – ICB – Second Cohort

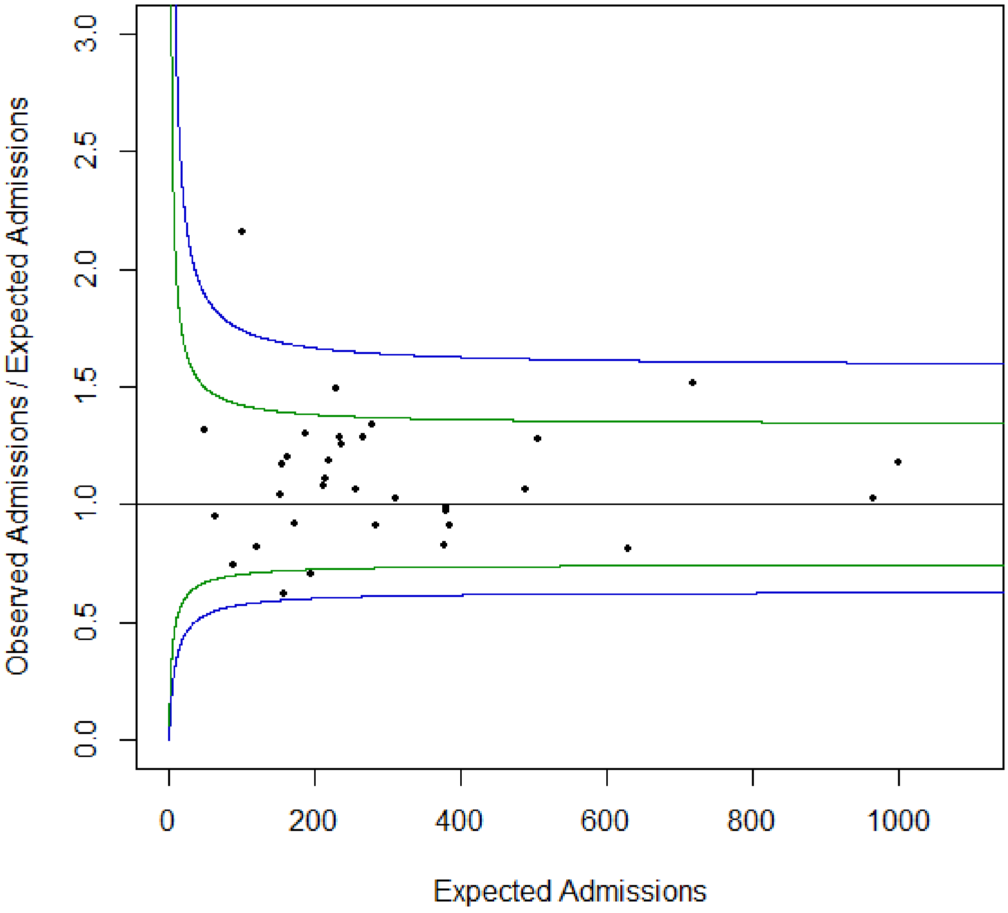

Hospital Admissions – ICB – Third Cohort

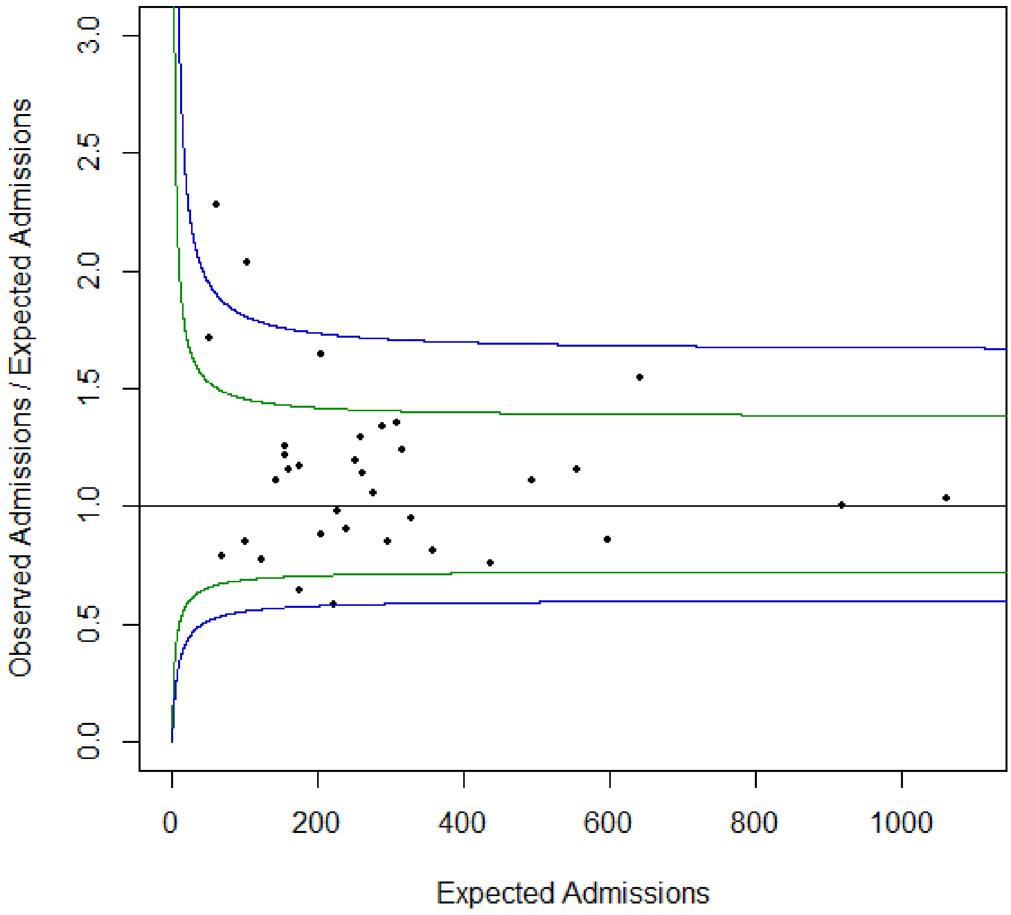

Hospital Admissions – ICB – Fourth Cohort

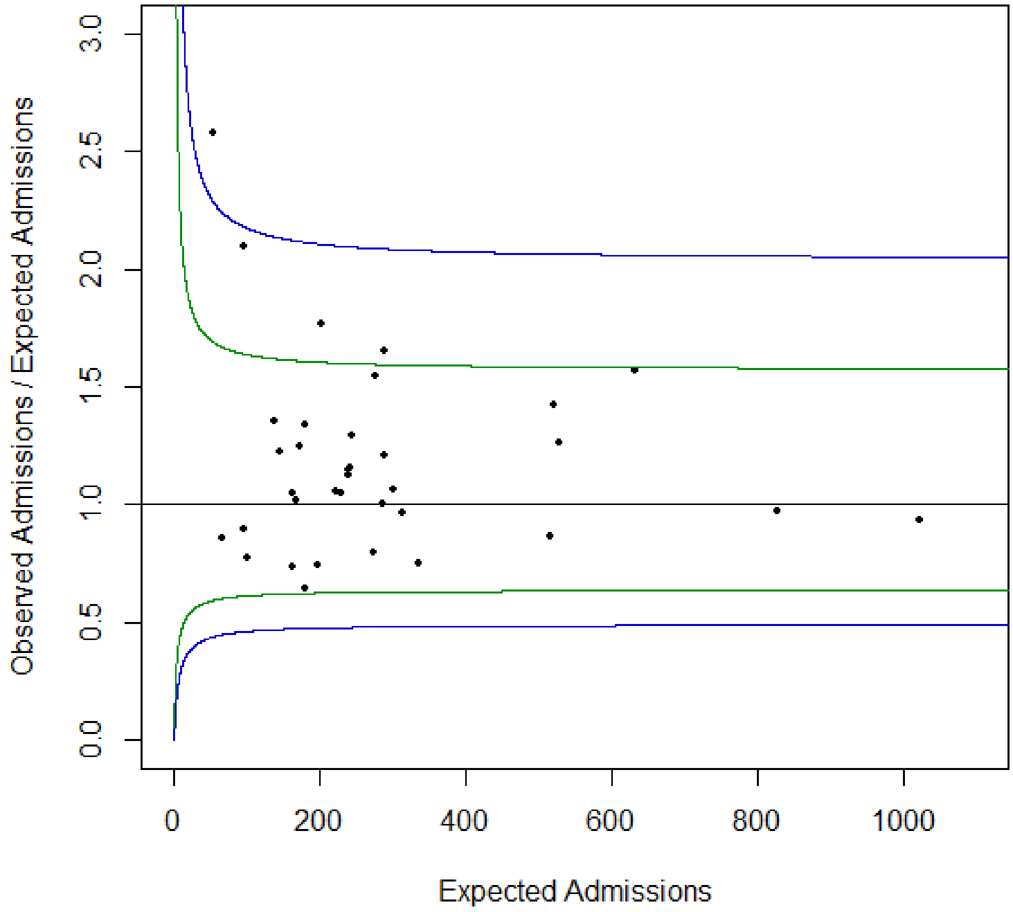

Supplement: Supplementary data [file bmjopen-16-5-s001.pdf]
